# Meta-analysis of quantitative trait loci for grain yield and component traits under reproductive-stage drought stress in an upland rice population

**DOI:** 10.1007/s11032-013-0012-0

**Published:** 2014-06-29

**Authors:** Kurniawan R. Trijatmiko, Supriyanta, Joko Prasetiyono, Michael J. Thomson, Casiana M. Vera Cruz, Sugiono Moeljopawiro, Andy Pereira

**Affiliations:** 1 Indonesian Center for Agricultural Biotechnology and Genetic Resources Research and Development, Jl. Tentara Pelajar 3A, Bogor, 16111 Indonesia; 2 Banguntapan Field Station, Faculty of Agriculture, Gadjah Mada University, Jl. Socio Justicia Bulaksumur, Yogyakarta, 55281 Indonesia; 3 International Rice Research Institute, DAPO Box 7777, Metro Manila, Philippines; 4 Crop, Soil and Environmental Sciences, University of Arkansas, Fayetteville, AR USA; 5 Virginia Bioinformatics Institute, Virginia Polytechnic and State University, Blacksburg, VA USA

**Keywords:** Recombinant inbred lines, Drought tolerance, Reproductive-stage drought, Grain yield, Spikelet fertility, Quantitative trait loci

## Abstract

A recombinant inbred population developed from a cross between high-yielding lowland rice (*Oryza sativa* L.) subspecies *indica* cv. IR64 and upland tropical rice subspecies *japonica* cv. Cabacu was used to identify quantitative trait loci (QTLs) for grain yield (GY) and component traits under reproductive-stage drought stress. One hundred fifty-four lines were grown in field trials in Indonesia under aerobic conditions by giving surface irrigation to field capacity every 4 days. Water stress was imposed for a period of 15 days during pre-flowering by withholding irrigation at 65 days after seeding. Leaf rolling was scored at the end of the stress period and eight agronomic traits were evaluated after recovery. The population was also evaluated for root pulling force, and a total of 201 single nucleotide polymorphism markers were used to construct the molecular genetic linkage map and QTL mapping. A QTL for GY under drought stress was identified in a region close to the *sd1* locus on chromosome 1. QTL meta-analysis across diverse populations showed that this QTL was conserved across genetic backgrounds and co-localized with QTLs for leaf rolling and osmotic adjustment (OA). A QTL for percent seed set and grains per panicle under drought stress was identified on chromosome 8 in the same region as a QTL for OA previously identified in three different populations.

**Electronic supplementary material:**

The online version of this article (doi:10.1007/s11032-013-0012-0) contains supplementary material, which is available to authorized users.

## Introduction

Rice (*Oryza sativa* L.) is particularly susceptible to water deficit compared to other crop species, and this sensitivity is especially severe around flowering (Lafitte et al. 2004). In South and Southeast Asia, more than 50 % of a total of 40 million ha rainfed lowland areas is affected by drought annually. These areas are found mainly in northeast Thailand, eastern India, Bangladesh, and Indonesia (Sarkarung and Pantuwan 1999). In northeast Thailand, yield reduction is estimated to be between 13 and 35 % (Jongdee et al. 1997). Drought during anthesis was found to be the most important of the top ten causes of yield losses in rainfed lowland rice in eastern India (Widawsky and O’Toole 1990).

Practical progress in breeding for drought tolerance in rice has been slow (Venuprasad et al. 2009). Drought tolerance therefore requires an analytical approach of dissecting and studying the contribution of different trait components using a quantitative trait locus (QTL)-based model. This approach is particularly suited to crops like rice for which dense genetic linkage maps are already available (Harushima et al. 1998; McCouch et al. 2002). Along with the availability of the map-based sequence of the rice genome (IRGSP 2005), the dense genetic linkage map allows rice molecular geneticists to narrow down the location of QTLs into a small region and predict the putative candidate genes in the region for gene cloning and validation by reverse genetics approaches (Hattori et al. 2009).

Many QTLs have been reported in rice for traits that are putatively associated with performance under drought, such as root system morphology (Champoux et al. 1995), osmotic adjustment (OA) (Robin et al. 2003), leaf membrane stability (Tripathy et al. 2000), and visual symptoms of leaf stress such as rolling and drying (Courtois et al. 2000). However, it is still unclear whether these secondary traits significantly contribute to grain yield (GY) under drought stress.

In recent years more attention has been given to mapping QTLs for GY and its components under managed stress environments (Babu et al. 2003; Lanceras et al. 2004; Yue et al. 2005; Vikram et al. 2011). Fukai et al. (1999) reported that flowering time is a major determinant of GY under late-season drought conditions in the rainfed lowland ecosystem. They also emphasized the ability of rice plants to maintain high leaf water potential as a trait relevant to stabilizing yield in rainfed rice planting areas.

In this study we tested the association between single nucleotide polymorphism (SNP) markers and GY under reproductive-stage drought stress using a recombinant inbred line (RIL) population derived from a cross between IR64 (a lowland *indica* cultivar) and Cabacu (an upland tropical *japonica* cultivar). We tried to minimize the effect of flowering time by choosing parental lines differing very little in flowering time and to minimize the remaining effect of flowering time by synchronizing the flowering date of the RIL population in the experiment. Our results showed that a region close to the *sd1* locus affecting gibberellic acid (GA) levels, which is associated with leaf rolling, has a significant effect on GY under drought stress. Comparison with three different populations showed that this region conferring GY under drought stress has been conserved across genetic backgrounds. The association between GA, abscisic acid (ABA), OA, spikelet fertility, and GY under drought stress were discussed.

## Materials and methods

### Plant material

The plant genotypes used here are the progeny of a cross between the cultivars IR64 and Cabacu (IRAT 177). IR64 is a lowland-adapted semi-dwarf *indica* cultivar from the International Rice Research Institute, Philippines. It is noted for high yield and a low level of drought avoidance, with shallow, thin roots. Cabacu is a tropical *japonica* cultivar from Brazil, with a comparatively thick, deep root system, and low but stable yields under drought stress in upland conditions. A total of 154 of F6 lines were produced by single seed descent (SSD).

### Screening for pre-flowering drought resistance

Lines were evaluated for pre-flowering drought resistance at the Banguntapan Field Station (latitude 7°48′40.56″S, longitude 110°24′30.69″E, altitude 100 m), grown on inceptisol soil type and located in Yogyakarta, Indonesia, during the 2004 dry season. The seeding date for each line was allocated to one of four planting dates, spaced at 4-day intervals. The latest maturing lines were assigned to the early date, and the earliest maturing lines were seeded at the latest. The objective was to synchronize the flowering time dates of all lines as closely as possible. The experiment was laid out in a randomized complete block design with two replications. The plot size was 3 rows × 20 hills (20 cm between rows and 20 cm between hills) with one plant per hill. All plots were surface irrigated to field capacity once every 4 days, except when water stress was imposed by withholding irrigation from 65 days after seeding (Babu et al. 2003, Lanceras et al. 2004). Fifteen days after withholding irrigation, leaf rolling scores (LRS) were made at midday on a 0–9 scale standardized for rice (IRRI 1996). After scoring, the stress was relieved and, thereafter, all plots were regularly irrigated until maturity. Ten plants from each plot were randomly chosen for the evaluation of eight traits: (1) days to heading (DTH), (2) plant height (PH), (3) panicles per plant (PPL), (4) spikelets per panicle (SPP), (5) grains per panicle (GPP), (6) percent seed set (PSS), (7) 100-grain weight (GW), and (8) grain yield per plant (GY).

### Screening for root pulling force

The lines were evaluated for root pulling force (RPF) at Banguntapan Field Station in the 2005 wet season. A randomized complete block design with two replications was used. The plot size was 4 rows × 11 hills (25 cm between rows and 25 cm between hills) with one plant per hill. All plots were surface irrigated to field capacity once every 4 days. Root pulling force was determined at the flowering stage (O’Toole and Soemartono 1981). Two hills per plot in each replication were randomly selected for determining the RPF.

### Evaluation of sensitivity to PEG-induced osmotic stress in the parents

Three-week-old seedlings were transferred from normal nutrient solution [without polyethylene glycol (PEG)] to one containing 20 % PEG 8000 (Capell et al. 2004). A non-stress plot remained in normal nutrient solution. Six days later, plant height was measured both for stress and non-stress conditions. Osmotic sensitivity index was calculated as the ratio of plant height reduction due to stress compared to the plant height under non-stress condition.

### SNP genotyping

Genomic DNAs of 154 RILs and two parents were isolated from leaves of 3-week-old seedlings using a chloroform-based DNA extraction protocol as described by McCouch et al. (1988). Approximately 2 µg of purified DNA at a concentration of 50 ng/µl was delivered to the Molecular Marker Applications Laboratory, International Rice Research Institute (Los Banos, Laguna, Philippines) for genotyping with a custom 384-plex single nucleotide polymorphism (SNP) set (RiceOPA2.1) evenly distributed in 12 rice chromosomes (Thomson et al. 2012). The genotyping procedure was performed as recommended in the GoldenGate Genotyping Assay for VeraCode Manual (Illumina VC-901-1001) as described previously (Thomson et al. 2012). The sample VeraCode Bead Plate was scanned immediately using default settings in the VeraScan software on the BeadXpress Reader System. Raw intensity data were analysed using the Genotyping module (v1.6.3) of the Illumina GenomeStudio (v2010.1) software. Genotype calling was performed using ALCHEMY software package (Wright et al. 2010).

### Statistical analysis

Simple statistics of each trait and correlation coefficient between the traits were computed using phenotypic means. The linkage map was constructed using the program MAPMAKER (Lander et al. 1987). The phenotypic and SNP genotypic data of 154 RILs were analyzed by composite interval mapping (CIM) using QGene v4.3.10 (Joehanes and Nelson 2008). Because *sd1* was detected in the RIL population with the largest effect on PH, we divided the RIL population into the *sd1* subpopulation (65 lines) and the *SD1* subpopulation (67 lines) based on the genotypes of id1022408 (genomic position: 37,298,115 bp) and id1023892 (genomic position: 39,545,238 bp) flanking *sd1* (genomic position: 38,381,339 bp) and performed QTL analysis for each subpopulation separately (Zhang et al. 2013). QTL names were designed following the standard rice QTL nomenclature (McCouch 2008). Construction of a consensus genetic map and QTL meta-analysis were performed using BioMercator v3.1 (Sosnowski et al. 2012).

## Results

### Analysis of grain yield parameters under drought

The recombinant inbred line (RIL) population was tested in a replicated field test for different yield-determining parameters under drought stress before flowering. During the experimental period (July–October) the average maximum temperature was 32.4 °C and the average minimum temperature was 23.1 °C, while the average relative humidity was 89.3 % at 7 a.m. and 66.0 % at 1 p.m. There was no rain throughout the experimental period. The mean and range of values for DTH, PH, PPL, SPP, GPP, PSS, GW, GY, RPF, and LRS in the RIL population are summarized in Table 1 and phenotype data are presented in Online Resource 1. Under the given drought stress, the GY of IR64 was similar to Cabacu with means of 17.5 g and 16.0 g per plant, respectively. In the RIL population, GY ranged from 1.4 to 24.3 g per plant and showed transgressive segregation in both directions, in which 91 % of the RIL population had GY less than both parents (Table 1 and Online Resource 2). Transgressive segregation in both directions was also observed for all other traits (Table 1 and Online Resource 2). The correlation coefficients between all traits were calculated and are shown in Table 2. GY under stress had higher correlation with yield components (GPP, PSS, GW, SPP, and PPL) than it did with primary trait (RPF), secondary trait (LRS), phenology (DTH), and plant-type trait (PH). RPF had positive correlations with GPP and PH. LRS had a positive correlation with DTH and negative correlations with PSS, GW, and GPP. There was no significant correlation between LRS and RPF.

**Table 1:** Phenotypic performance of parents and 154 recombinant inbred lines

Traits	Parental lines			RILs			
	IR64 mean	Cabacu mean	IR64 versus Cabacu^a^	Mean	Range	CV^b^	*H* (%)^c^
Leaf rolling score (LRS)	2.2	0.6	**	2.7	0.0–8.0	27.7	92.0
Root pulling force (RPF)	11.4	19.0	**	17.4	6.0–31.8	36.7	41.5
Grain yield per plant (GY)	17.5	16.0	ns	9.1	1.4–24.3	44.2	64.1
Panicles per plant (PPL)	18.7	11.5	**	13.2	5.3–34.8	30.1	63.7
Spikelets per panicle (SPP)	82.5	103.3	**	84.7	37.9–165.4	21.8	59.0
Grains per panicle (GPP)	66.6	88.6	**	51.9	5.8–146.7	38.5	64.1
Percent seed set (PSS)	85.1	88.8	ns	61.3	9.0–91.8	28.0	73.1
100-grain weight (GW)	2.2	3.1	**	2.2	1.5–3.1	15.1	53.3
Days to heading (DTH)	99.0	109.0	**	97.0	82.0–114.0	4.0	83.5
Plant height (PH)	65.5	97.6	**	77.1	45.6–132.5	8.5	92.8

*ns* not significant

** Significant at *P* < 0.01

^a^Difference between two parents

^b^Coefficient of variation

^c^H^2^ estimates calculated on a family mean basis

**Table 2:** Phenotypic correlations for GY and other traits

Trait	PPL	SPP	PSS	GPP	GW	DTH	PH	RPF	LRS
GY	0.219*	0.237*	0.600**	0.627**	0.320**	–	–0.169	–	–
PPL		–	–0.281**	–0.256*	–	0.177	–0.306**	–	–
SPP			–	0.515**	–	–	0.338**	–	–
PSS				0.815**	0.333**	–0.269**	–	–	–0.198
GPP					0.267**	–0.215*	–	0.184	–0.160
GW						–0.269**	–	–	–0.196
DTH							–	–	0.329**
PH								0.163	–
RPF									–

All correlations shown are significant at *P* < 0.05

*PPL* panicles per plant, *SPP* spikelets per panicle, *PSS* percent seed set, *GPP* grains per panicle, *GW* 100-grain weight, *DTH* days to heading, *PH* plant height, *RPF* root pulling force, *LRS* leaf rolling score, *GY* grain yield per plant

* Significant at *P* < 0.01

** Significant at *P* < 0.001

*LRS* leaf rolling score, *RPF* root pulling force, *GY* grain yield, *PSS* percent seed set, *GPP* grains per panicle, *SPP* spikelets per panicle, *DTH* days to heading, *PH* plant height

### Segregation analysis of SNP markers in the RIL population

The parents of the population were screened for SNPs using an Illumina 384-plex SNP set (RiceOPA2.1), showing 52 % polymorphism between them (Online Resource 3). This was comparable to the previous results using the same SNP set on the parental lines of two other *indica*/*japonica* mapping populations, 93-11/Nipponbare and IR64/Moroberekan, i.e. 52 % and 50 %, respectively (Thomson et al. 2012). Although this was lower than our previous simple sequence repeat survey, which showed 61 % polymorphism (data not shown), the RiceOPA2.1 provided 201 polymorphic and well-distributed SNP markers for the IR64/Cabacu population, with average distance between markers of 7.9 cM (Online Resource 4). Without selection, the expected genotypic ratio in the SSD F6 generation would be 48.4:48.4:3.1 for the homozygous IR64, homozygous Cabacu, and heterozygous IR64/Cabacu genotype classes (or 50 % IR64 alleles to 50 % Cabacu alleles). Out of 201 marker loci, 23.4 % (47 markers) were skewed toward one or the other parent: 21.9 % (44 markers) showed overrepresentation of IR64 alleles, while 1.5 % (3 markers) were skewed toward Cabacu (*P* < 0.01). This high frequency of distorted markers was not surprising considering previous work showing that RIL populations had significantly higher frequencies of distorted marker segregation (39.4 ± 2.5 %) than other population structures, comparing 53 populations across 30 species (Xu et al. 1997). The segregation distortion in the IR64/Cabacu population was observed in nine chromosomes, varying from only one marker on chromosomes 1, 4, and 12–13 markers on chromosome 7 (Online Resource 5). Some of the distorted markers were randomly distributed along the chromosomes, but most occurred in clusters, and in chromosome 7 the marker distortion extended to the whole short arm (Online Resource 5). Comparison of the distorted regions in the current study with those in the previous studies revealed several regions in common (Online Resource 6). Six chromosomal regions (chromosomes 1, 3, 4, 7, and 11) were located at or near previously reported gametophytic gene loci (*ga*) and/or sterility loci (*S*) associated with distorted segregation (Online Resource 6).

### QTL analysis of grain yield under drought

**Fig. 1:**
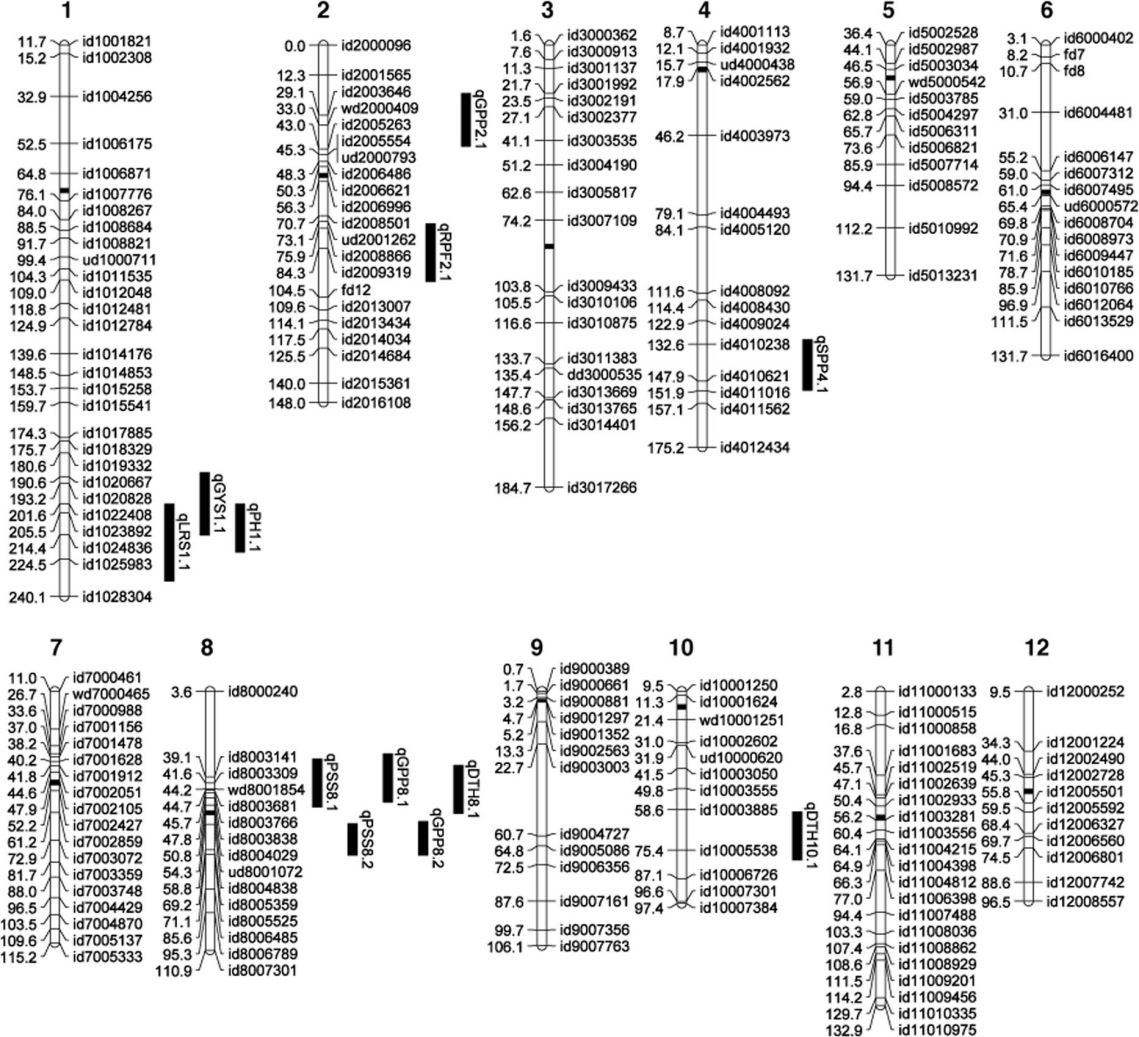
Mapping of QTLs for grain yield (GY) and component traits under reproductive drought in an IR64/Cabacu population: molecular linkage map of an IR64 × Cabacu mapping population constructed with 201 SNP markers. The position of the significant QTLs are illustrated by *black bars* next to the chromosomes. Centromeres are shown as *black boxes*

Composite interval mapping was used to dissect a total of 12 QTLs from the phenotypic data, located on chromosomes 1, 2, 4, 8, and 10 (Fig. 1; Table 3). The contributions of the 12 QTLs to the phenotypic variation ranged from 8.8 to 38.6 %, and their LOD scores ranged from 3.07 to 16.30. One QTL was detected for GY under drought stress, located on the long arm of chromosome 1. This QTL explained 9.1 % of GY variation, in which IR64 contributed the favorable allele. This QTL co-localized with QTLs for LRS (*R* ^2^ = 9.1 %) and PH (*R* ^2^ = 38.6 %), in which IR64 alleles had beneficial effects. A QTL for RPF was detected on chromosome 2 (*R* ^2^ = 10.0 %), in which Cabacu contributed the favorable alleles. Two QTLs for PSS were detected on chromosome 8 (*R* ^2^ = 14.7 and 20.5 %), both co-localized with QTLs for GPP (*R* ^2^ = 11.2 and 16.4 %), in which IR64 contributed the favorable alleles. One of these QTLs co-localized with a QTL for DTH (*R* ^2^ = 8.8 %), with the IR64 allele decreasing the DTH. Another QTL for GPP was detected on chromosome 2 (*R* ^2^ = 8.9 %), in which Cabacu contributed the favorable allele. A QTL for DTH was detected on chromosome 10 (*R* ^2^ = 11.0 %), in the same region as the previously identified *Early heading date 1* (*Ehd1*) gene (Doi et al. 2004). A QTL for SPP was detected in the long arm of chromosome 4 (*R* ^2^ = 10.6 %), in the same region as the recently identified *Spike* (*Nal1*) gene controlling spikelet number (Fujita et al. 2013), in which the tropical *japonica* parent contributed the favorable allele.

**Table 3:** QTLs for leaf rolling score, root pulling force, yield, and yield components under drought condition identified from the IR64/Cabacu population

Traits	QTLs	Chr^a^	Peak marker	Increased effect	LOD^b^	*R* ^2^ (%)^c^	A^d^
Leaf rolling score	*qLRS1.1*	1	id1025983	Cabacu^e^	3.2^f^	9.1	–0.7
Root pulling force	*qRPF2.1*	2	id2009319	Cabacu	3.5	10.0	–2.2
Yield per plant	*qGY1.1*	1	id1023892	IR64	3.2	9.1	1.5
Percent seed set	*qPSS8.1*	8	id8003838	IR64	**5.3** ^g^	14.7	9.2
	*qPSS8.2*	8	id8005359	IR64	**7.7**	20.5	11.3
Grains per panicle	*qGPP2.1*	2	wd2000409	Cabacu	3.1	8.9	–7.6
	*qGPP8.1*	8	id8003838	IR64	**4.0**	11.2	8.2
	*qGPP8.2*	8	id8005359	IR64	**6.0**	16.4	10.8
Spikelets per panicle	*qSPP4.1*	4	id4010621	Cabacu	**3.7**	10.6	–8.7
Days to heading	*qDTH8.1*	8	id8004029	Cabacu	3.1	8.8	–2.1
	*qDTH10.1*	10	id10005538	Cabacu	**3.9**	11.0	–2.7
Plant height	*qPH1.1*	1	id1024836	Cabacu	**16.3**	38.6	–12.3

^a^Chromosome no

^b^Logarithm of odds score

^c^Relative contributions of the putative QTLs to the phenotypic variation

^d^Additive effect. A positive or negative value indicates that the allele from IR64 or Cabacu increases the trait value, respectively

^e^A higher score for LRS is unfavorable (more leaf rolling)

^f^QTLs in *regular type* were identified at *P* < 0.05 using permutation analysis

^g^QTLs in bold face were identified at *P* < 0.01

### Meta-analysis of QTLs for grain yield parameters and drought response

**Fig. 2:**
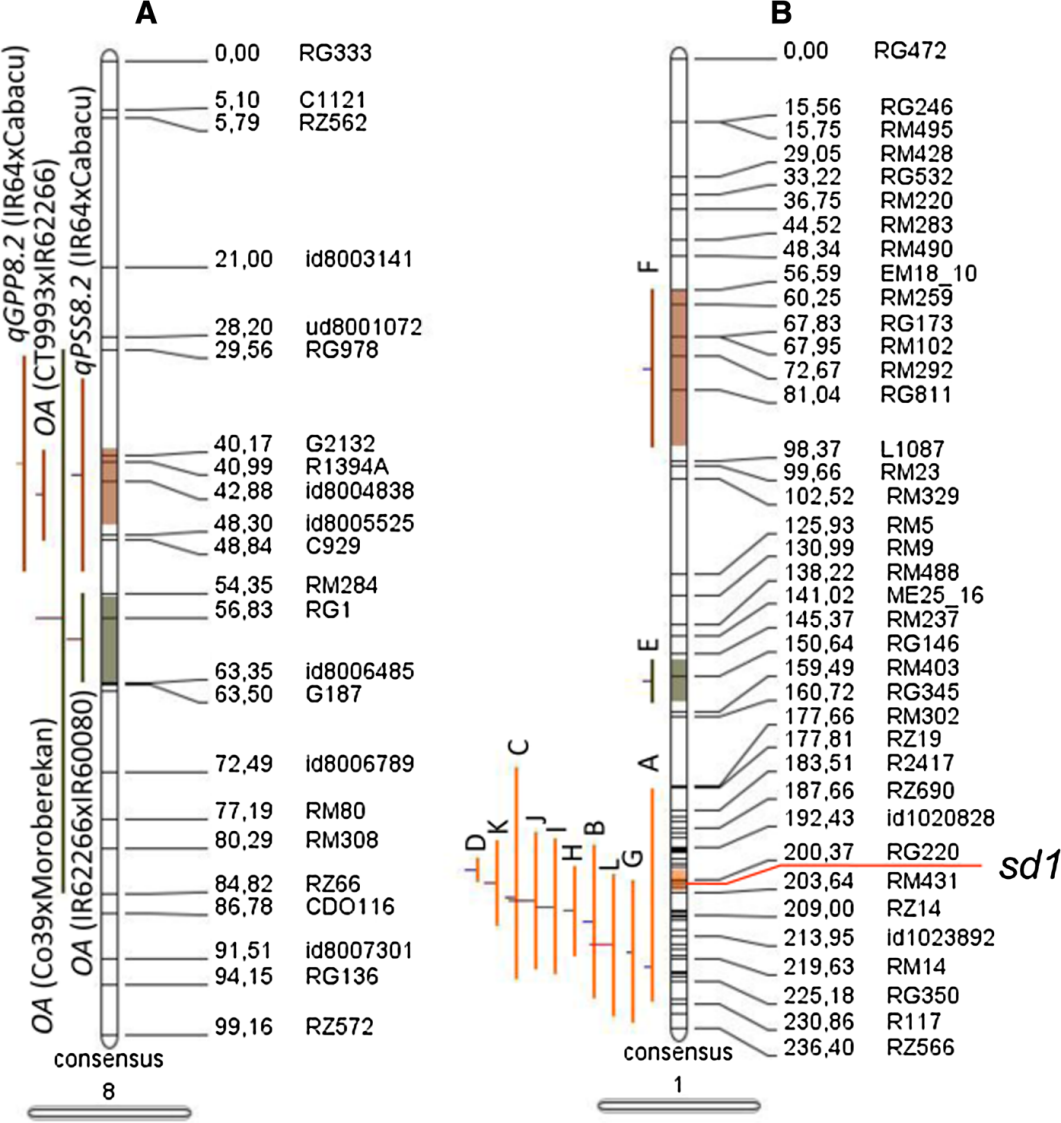
Genomic regions of rice chromosomes showing co-localization of QTLs. Construction of consensus genetic map and QTL meta-analysis were performed using BioMercator v3.1 (Sosnowski et al. 2012). **a** Rice chromosome 8 showing QTLs for grains per panicle (GPP), percent seed set (PSS), and osmotic adjustment (OA) across rice genetic backgrounds. The QTLs *qGPP8.2* (IR64/Cabacu) and *qPSS8.2* (IR64/Cabacu) were from Fig. 1 of this study, *OA* from the mapping population Co39/Moroberekan (Lilley et al. 1996), *OA* from IR62266/IR60080 (Robin et al. 2003), and *OA* from CT9993/IR62266 (Nguyen et al. 2004). **b** Rice chromosome 1 showing the common QTLs for grain yield (GY), leaf rolling (LR), osmotic adjustment (OA) and plant height (PH) under drought stress across rice genetic backgrounds. The QTLs shown are (*A*) *GY* from IR64/Cabacu from Fig. 1 of this study, (*B*) *GY* from N22/MTU1010 (Vikram et al. 2011), (*C*) *GY* from IR64/Azucena (Lafitte et al. 2002), (*D*) *GY* from CT9993/IR62266 (Kumar et al. 2007), (*E*) *GY* from Zhenshan97/IRAT109 (Yue et al. 2005), and (*F*) *GY* from CT9993/IR62266 (Babu et al. 2003); (*G*) *LR* from IR64/Cabacu from Fig. 1 of this study; (*H*) *LR* from Bala/Azucena (Price et al. 2002), and (*I*) *LR* from IR64/Azucena (Courtois et al. 2000); (*J*) *OA* from Co39/Moroberekan (Lilley et al. 1996) and (*K*) *OA* from IR62266/IR60080 (Robin et al. 2003); and (*L*) *PH* from IR64/Cabacu from Fig. 1 of this study

The QTLs identified in this study were compared with other published loci for GY parameters and drought response using BioMercator v3.1 (Sosnowski et al. 2012). The *qPSS8.2* and *qGPP8.2* loci identified in the present study were located in the same genomic region as the QTL for OA detected previously (Robin et al. 2003; Lilley et al. 1996; Nguyen et al. 2004) (Fig. 2a). The *indica* lowland parental lines contributed the favorable alleles for PSS, GPP, and OA.

In a comparison of QTLs for GY under stress, the *qGY1.1* locus identified in the present study was located in the same genomic region as the QTL for GY under stress detected in other populations (Kumar et al. 2007; Vikram et al. 2011) (Fig. 2b). The semi-dwarf gene *sd1* (Huang et al. 1996), which affects many agronomic traits (Xia et al. 1991; Zhang et al. 2013), is located in this region. A QTL for GY under aerobic growth conditions has been previously mapped in this region in a population derived from a cross between IR64 and Azucena (traditional upland *japonica* cultivar) in four different experiments, in which the IR64 allele consistently gave positive effects to yield (Lafitte et al. 2002) (Fig. 2b). This region on rice chromosome 1 is homeologous with a segment of barley chromosome 3H where a single locus putatively associated with GY was identified (Cuesta-Marcos et al. 2009).

The QTL for leaf rolling, *qLRS1.1*, identified in the present study was located in the same genomic region in other populations as the QTLs for LRS (Courtois et al. 2000; Price et al. 2002) and OA (Robin et al. 2003; Lilley et al. 1996) (Fig. 2b). The semi-dwarf gene *sd1* is located in this region, and in all cases the semi-dwarf parental lines contributed the favorable alleles for leaf rolling and OA.

## Discussion

### Grain yield component variation and correlation

GY under drought stress is a function of yield potential and drought resistance mechanisms of crop plants. These two factors should be considered in any attempt to study the genetic mechanisms controlling GY under drought stress. Flowering time is a major determinant of GY and its components under drought stress, especially for late-season drought (Fukai et al. 1999), probably working through a drought escape mechanism (Xu et al. 2005). We therefore used parents differing little in flowering time and tried to minimize the remaining effect of flowering time by synchronizing the flowering date of the RIL population in the experiment by staggered planting. We examined the GY along with its components and two putative drought-related traits, i.e. RPF and LRS. All phenotypes showed transgressive variation in both directions, indicating that the two parental lines contribute favorable alleles for all the traits.

Aerobic rice is a cultivation method that conserves water use and would therefore be useful in water-scarce regions and periods. This condition is also susceptible to drought at specific stages and the stability of crop production depends on the resistance of the rice crop at various stages during its life cycle. In this study, we provided aerobic conditions by giving surface irrigation to field capacity once every 4 days, with a period of drought stress during pre-flowering stage. Irrigation was withheld for 15 days during the period 30–15 days before the average flowering date of the RIL population. There was no rain throughout the experiment period. Under this aerobic soil with mild drought stress condition, the GY of the flood-cultivation-adapted parent (IR64) decreased significantly (50 % decrease in comparison to irrigated condition, data not shown). More than 90 % of the RIL population showed GY less than either parental line, and in some cases very low absolute values (Online Resource 2). This could be due to segregation of unfavorable alleles from both parents or also perhaps an indication of hybrid breakdown (sterility and weakness in F2 or later generations) commonly seen in crosses between *indica* and *japonica* varieties (Oka 1988). More studies are needed to distinguish the basis of lower GY in the populations. However, the recovery of progeny lines with higher GY under drought than parents showed promise for improving upland rice for high yield with QTL from the high-yielding lowland cultivar IR64.

In this analysis, the correlation of GY under drought stress was predominantly determined by its components, i.e. grains per panicle (GPP) (*r* = 0.627), percent seed set (PSS) (*r* = 0.600), 100-grain weight (GW) (*r* = 0.320), spikelets per panicle (SPP) (*r* = 0.237), and panicles per plant (PPP) (0.219). These results support the concept that an important criterion for obtaining stable and high yield of rainfed lowland rice under drought conditions is to have high potential yield under well-watered conditions (Fukai et al. 1999). In addition, the most significant traits related to GY (GPP and PSS) are the capacity of plants to maintain the fertility of spikelets during drought stress, and this might be related to their ability to maintain high leaf water potential by extracting more water from deep soil layers or to maintain cell turgor pressure despite low leaf water potential by OA. In this study, RPF showed positive correlations with GPP (*r* = 0.184). On the other hand, LRS as a negative indicator of OA (see below) showed negative correlations with PSS (−0.198) and GPP (−0.160).

It is important to note that the grain yield data obtained in this study reflects a combination of the yield potential and the ability to maintain yield under stress. Further research with the inclusion of an unstressed control will enable the normalization of the grain yield under stress for the confounding factors by calculating the residuals of the multiple regression of yield under stress on yield under non-stress and days to heading under non-stress, as suggested by Bidinger et al. (1982).

### Co-localization of QTLs for PSS, GPP, and OA

Two QTLs for PSS were identified in this study on chromosome 8. One of these QTLs co-localized with QTLs for GPP in this study and QTLs for OA in two other populations. This region on rice chromosome 8 is syntenic with a region on the short arm of wheat chromosome 7 where an osmoregulation locus (*or*) was identified (Zhang et al. 2001). This QTL co-localization might indicate the important role of OA in spikelet fertility during a water deficit period. OA results in a decrease in the cell osmotic potential and thus in maintenance of water absorption and cell turgor pressure (Serraj and Sinclair 2002), which might contribute to maintenance of stomatal opening, allowing a continuous supply of carbohydrates to spikelets and higher enzyme activities (Sheoran and Saini 1996) which might assist fertilization and reduce spikelet sterility (Saini and Lalonde 1998).

### Co-localization of QTLs for PH, OA, LRS, and GY under reproductive-stage drought stress

A QTL for GY under drought stress was characterized here at the *sd1* locus region. This QTL was consistently identified across diverse genetic backgrounds (Fig. 2b). QTLs for PH and LRS also co-localized in this region. Interestingly, a QTL for OA was previously identified in the same region in two different populations (Fig. 2b). In all cases, the semi-dwarf parental lines contributed the favorable alleles.

The co-localization of QTLs for several traits can be due to the pleiotropic action of a single gene or multiple linked genes, each affecting one trait (Studer and Doebley 2011). Co-localization of QTLs for GY under drought stress and the *sd1* gene in rice has been previously suggested due to linkage rather than pleiotropy (Kumar et al. 2007). This hypothesis cannot be tested in primary mapping populations because there is always a good possibility that the QTL is not located precisely at the maximum likelihood position and typical approximate confidence intervals for QTL positions are in the order of 20 cM (Holland 2004). However, our current result showing that the semi-dwarf parental line (IR64) contributed the favorable alleles for PH, LRS and GY allows us to speculate that the co-localization arose because of pleiotropic action of the *sd1* gene.

Accumulating evidence for pleiotropy stems from the molecular drought tolerance literature. A microarray study showed that there is an antagonistic regulation of almost all ABA-responsive and GA-responsive genes by both hormones in rice (Yazaki et al. 2004). In addition, another study showed that ABA enhances IVR2 vacuolar invertase activity and expression in maize (Trouverie et al. 2003). The reduced level of GA in genotypes that contain the *sd1* allele (Spielmeyer et al. 2002) may have a beneficial effect through the activation of invertase by ABA, which allows hydrolysis of sucrose for rapid accumulation of glucose and fructose in leaves of drought-stressed plants, with positive impact on OA. It has been shown that OA delays leaf rolling to lower leaf water potentials, thus maintaining the effective leaf area for light interception and the diffusion of CO_2_, both of which sustain photosynthesis (Hsiao et al. 1984).

It has been shown that, in general, the tropical *japonica* rice varieties have low OA, while *indica* rice varieties have higher OA (Lilley and Ludlow 1996). Variety 63–83 was shown to be the sixth worst variety of 61 tested while IR64 was the 17th best. The tropical *japonica* parent in this study, Cabacu (IRAT 177), is a spontaneous mutant selected from IRAT 79 (Châtel and Guimaraes 2002), while IRAT 79 is a gamma-ray mutant of 63–83. Our glasshouse experiment using PEG showed that the osmotic sensitivity index of IR64 and Cabacu was 0.067 ± 0.057 and 0.245 ± 0.020, respectively (data not shown), in which the higher index indicates more sensitivity to osmotic stress imposed by PEG.

It is interesting to note that in the IR64/Azucena doubled haploid population, the effect of the IR64 allele in the *sd1* region on yield in aerobic fields was clearly positive, but there was no effect of this allele on yield in lowland environments (Lafitte et al. 2002). A recent study showed a pleiotropic effect of the loss-of-function (*sd1*) allele on the spikelet fertility in this population across eight environments (Zhang et al. 2013; Online Resource 8). Although these findings in the mapping and molecular studies support the idea of pleiotropy, more studies are needed to prove it. The creation of near- isogenic lines in which the *sd1* gene is fixed for the IR64 haplotype, but the regions flanking it are segregating for IR64 versus Cabacu chromosomal segments, will enable us to verify whether tight linkage or pleiotropy is causing the co-localization (Studer and Doebley 2011).

### Implication for breeding for rainfed systems

We divided the IR64/Cabacu RIL population into two groups following the strategy of Zhang et al. (2013), i.e. *SD1* subpopulation and *sd1* subpopulation, performing QTL analysis separately, and identified three categories: the *SD1*-mediated, *SD1*-repressed, and *SD1*-independent QTLs. The *SD1*-mediated QTLs comprised those QTLs that were detected only in the whole population and the *SD1* subpopulation, but not in the *sd1* subpopulation. The *SD1*-repressed QTLs comprised those QTLs that were detected only in the whole population and the *sd1* subpopulation, but not in the *SD1* subpopulation. The QTLs detectable in the whole and both the *SD1* and *sd1* subpopulations belonged to the *SD1*-independent category. We found that in the *SD1* subpopulation, in which the functional *SD1* gene is fixed, 66 % of the subpopulation had plant height in between IR64 and Cabacu (Online Resource 7), indicating the involvement of dwarfing genes other than *sd1* contributing to the quantitative variation in PH (Online Resource 8). Our study also showed that QTLs *qGPP8.2* for GPP and *qPSS8.2* for PSS under drought stress, in which IR64 contributed the favorable alleles, were detectable only in the whole population and *SD1* subpopulation, but not in the *sd1* subpopulation (Online Resource 8), indicating that these QTLs are mediated by the functional *SD1* gene (Zhang et al. 2013). While adding new genes for ‘green’ traits into the current high-yielding semi-dwarf rice cultivars has been suggested to achieve the Green Revolution II (Khush 2001), this strategy should be more effective for high-input irrigated systems and/or the short-day season of the tropics where the *SD1*-repressed system tends to express more strongly (Zhang et al. 2013). For low-input rainfed systems, in which most semi-dwarf rice cultivars show poor adaptability, partially attributable to the association of *sd1* with reduced root length (Zhang et al. 2013), an alternative strategy to develop new varieties with high nutrient use efficiency and better abiotic-stress tolerance while keeping *SD1* should be applied. The QTL for PSS (spikelet fertility) we identified in this study, which is mediated by the functional *SD1* gene (gibberellin 20-oxidase), will be useful in breeding programs to develop superior rice cultivars that have better drought-stress tolerance with different heights suitable for different low-input rainfed environments. Percent seed set has been judged the most practical character by which to score cultivar performance under drought stress at reproductive phase (Garrity and O’Toole 1994), and therefore a useful component trait to select for.

This study demonstrates that meta-analysis of QTLs across diverse populations enables identification of consensus loci for GY and component traits under drought on chromosomes 1 and 8 mapped in this study and other published works. QTLs *qGPP8.2* for GPP and *qPSS8.2* for PSS under drought stress, identified in this study, co-localized with QTLs for OA in two other populations, which indicates the important role of OA in spikelet fertility during water deficit periods. This conserved region provides a primary target for fine-structure mapping and candidate gene identification activity, which can be facilitated by genome and transcriptome sequencing (RNA-Seq) and further validation with reverse genetics approaches.

## Electronic supplementary material

Below is the link to the electronic supplementary material.

Table presents phenotype data of 154 recombinant inbred lines (IR64/Cabacu). (XLSX 34 kb)

Phenotypic distribution of drought stress responses under pre-flowering drought stress in a RIL population derived from a cross between IR64 and Cabacu. (PDF 664 kb)

Table presents SNP genotype data of 154 recombinant inbred lines (IR64/Cabacu) (XLS 461 kb)

Table presents the distribution of polymorphic SNP markers on 12 rice chromosomes observed in this study. (PDF 69 kb)

Segregation distortion detected along the 12 rice chromosomes. The chromosomal position in centimorgans is on the x-axis, and LOG(0.01/*P*) values are on the y-axis. A value of LOG(0.01/*P*)>0 corresponds to *P*< 0.01. (PDF 315 kb)

Table presents the comparative study of segregation distortion. (PDF 77 kb)

Frequency distribution of plant height in the *SD1* subpopulation of the IR64/Cabacu RIL population. (PDF 143 kb)

Table presents QTLs for percent seed set, grains per panicle and plant height under drought condition identified in the IR64/Cabacu RIL population and subpopulations with fixed alleles at the *sd1* locus (PDF 80 kb)
