# Supplementary material for: Meta-analysis of quantitative trait loci for grain yield and component traits under reproductive-stage drought stress in an upland rice population
Source: Mol Breed. 2014 Jun 29;34(2):283–95. doi: 10.1007/s11032-013-0012-0 (PMC4092238; doi:10.1007/s11032-013-0012-0)

Online Resource 2 Molecular Breeding

Meta-analysis of QTLs for grain yield and component traits under reproductive-stage drought stress in an upland rice population.

Kurniawan R. Trijatmiko, Supriyanta, Joko Prasetiyono, Michael J. Thomson, Casiana M. Vera Cruz, Sugiono Moeljopawiro, Andy Pereira*.

*Crop, Soil & Environmental Sciences, University of Arkansas, Fayetteville, AR, USA; *apereira@uark.edu

Online Resource 2

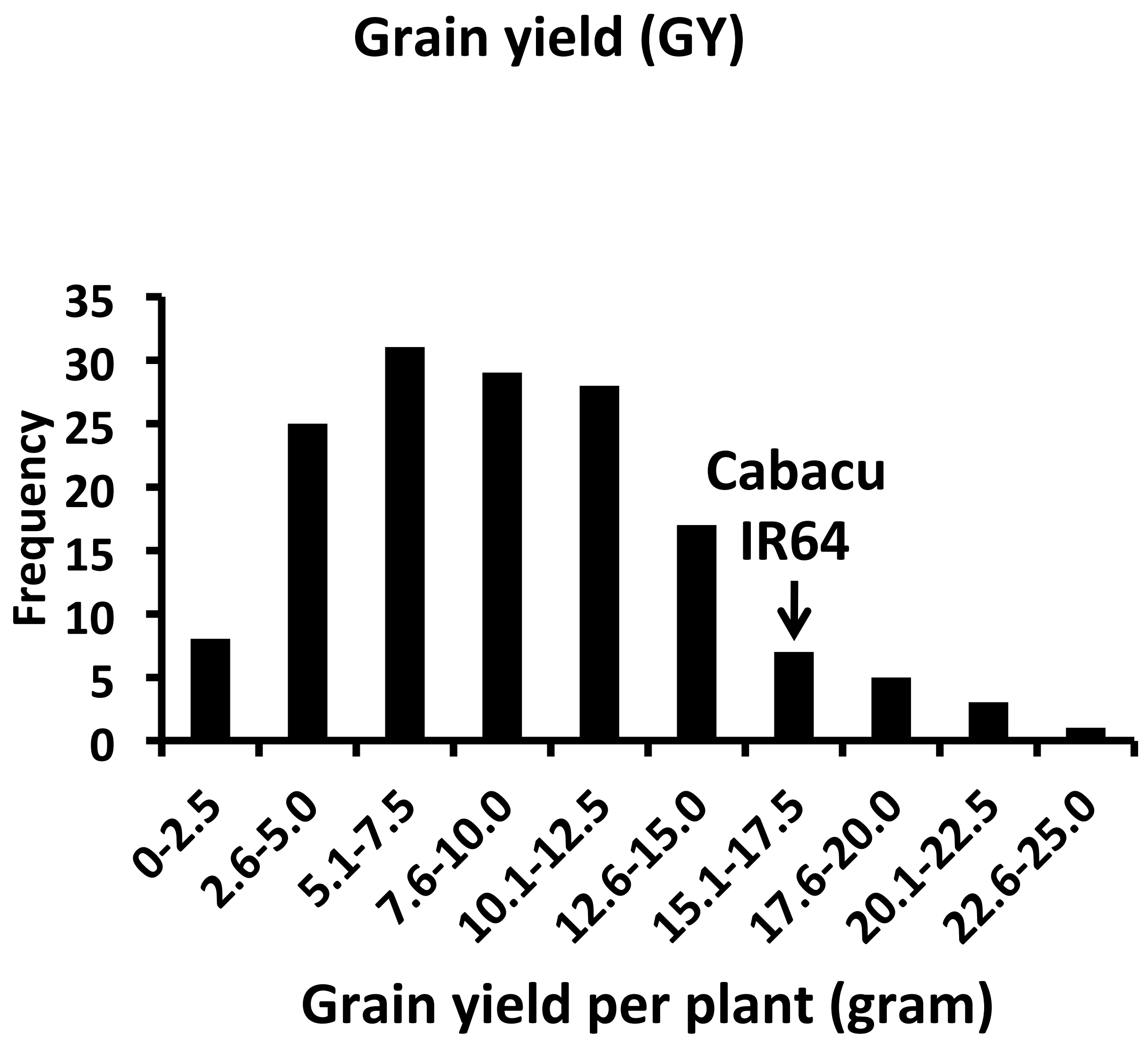

Online Resource 2

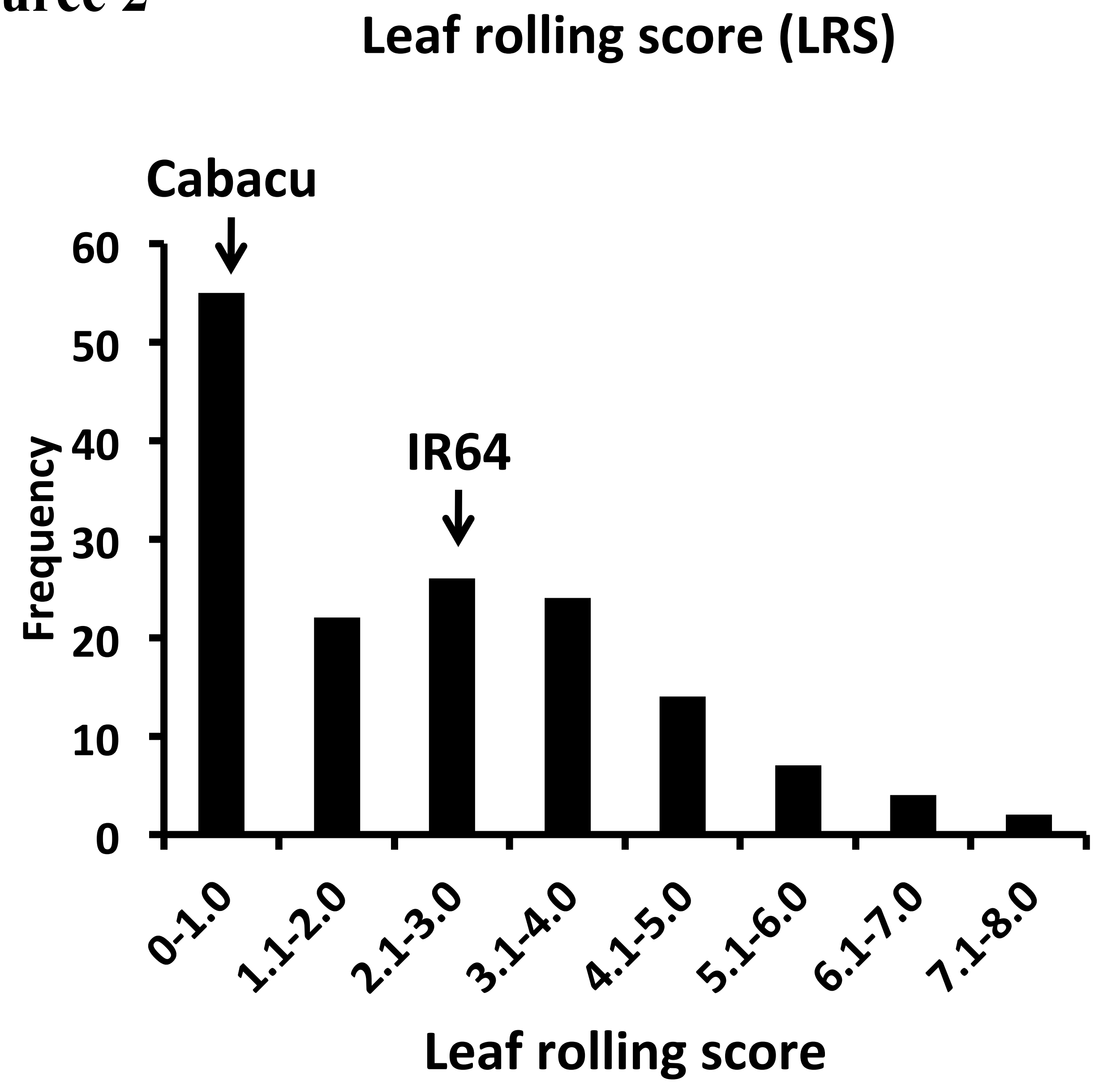

Online Resource 2

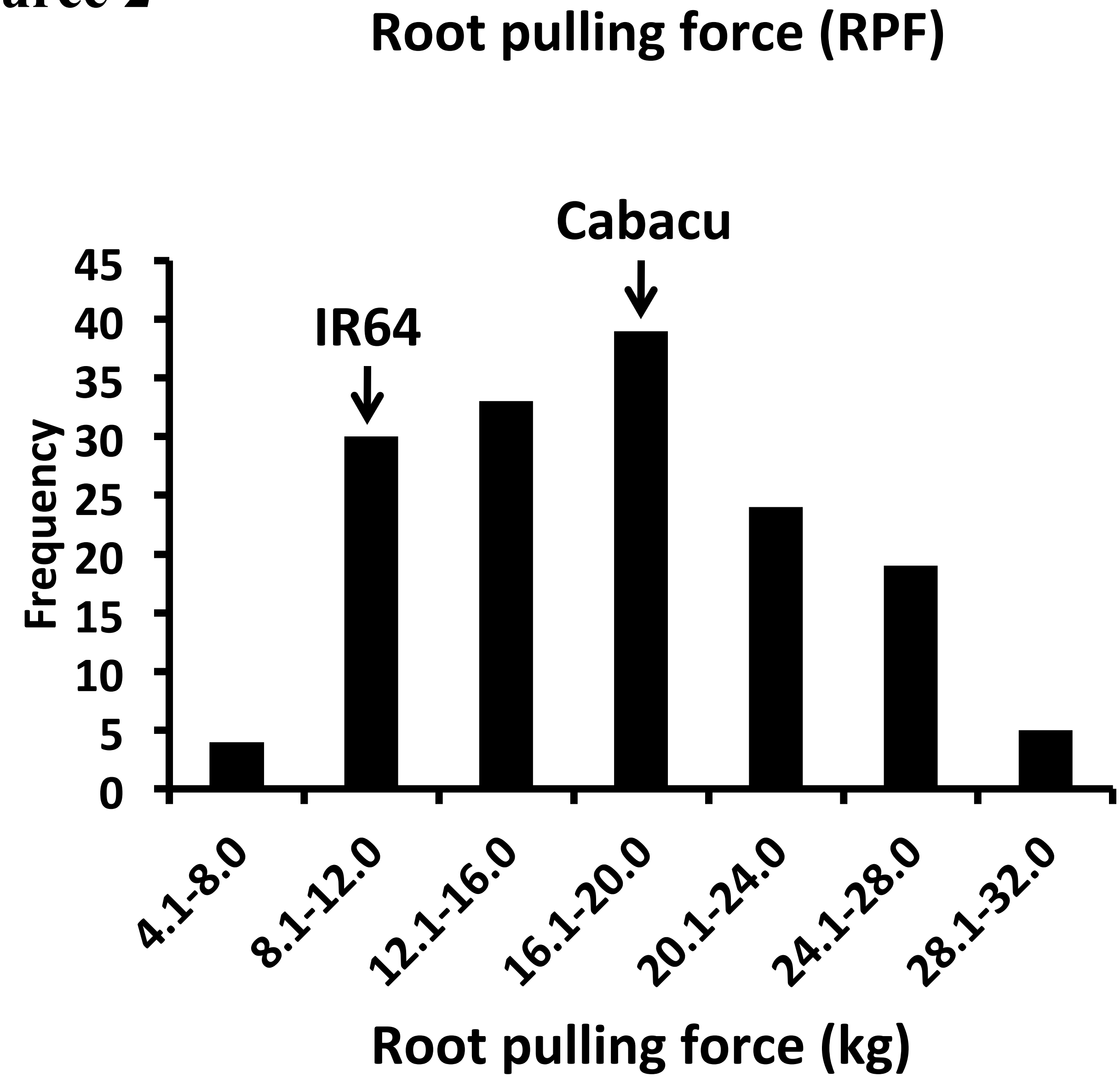

Online Resource 2

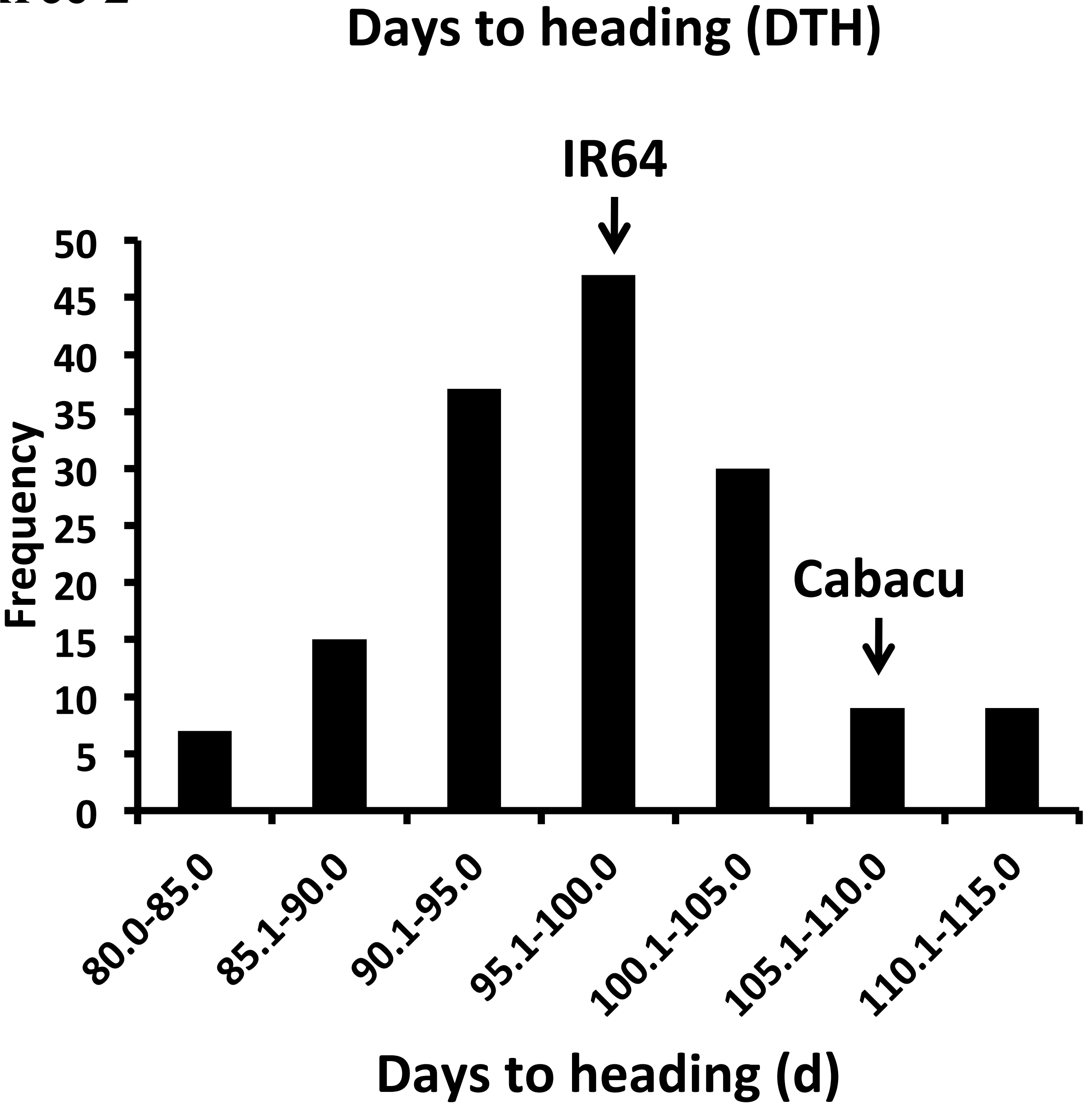

Online Resource 2

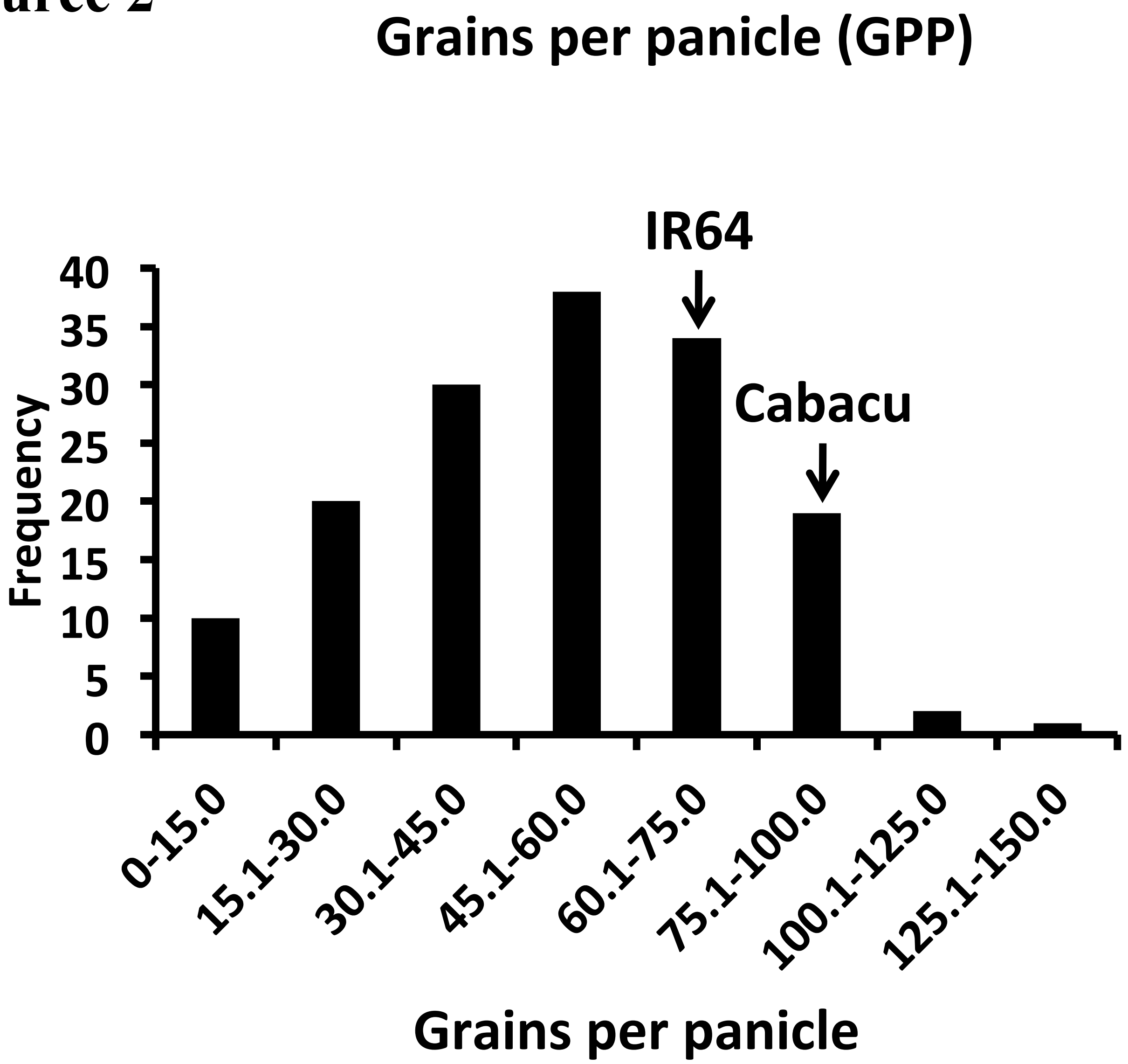

Online Resource 2

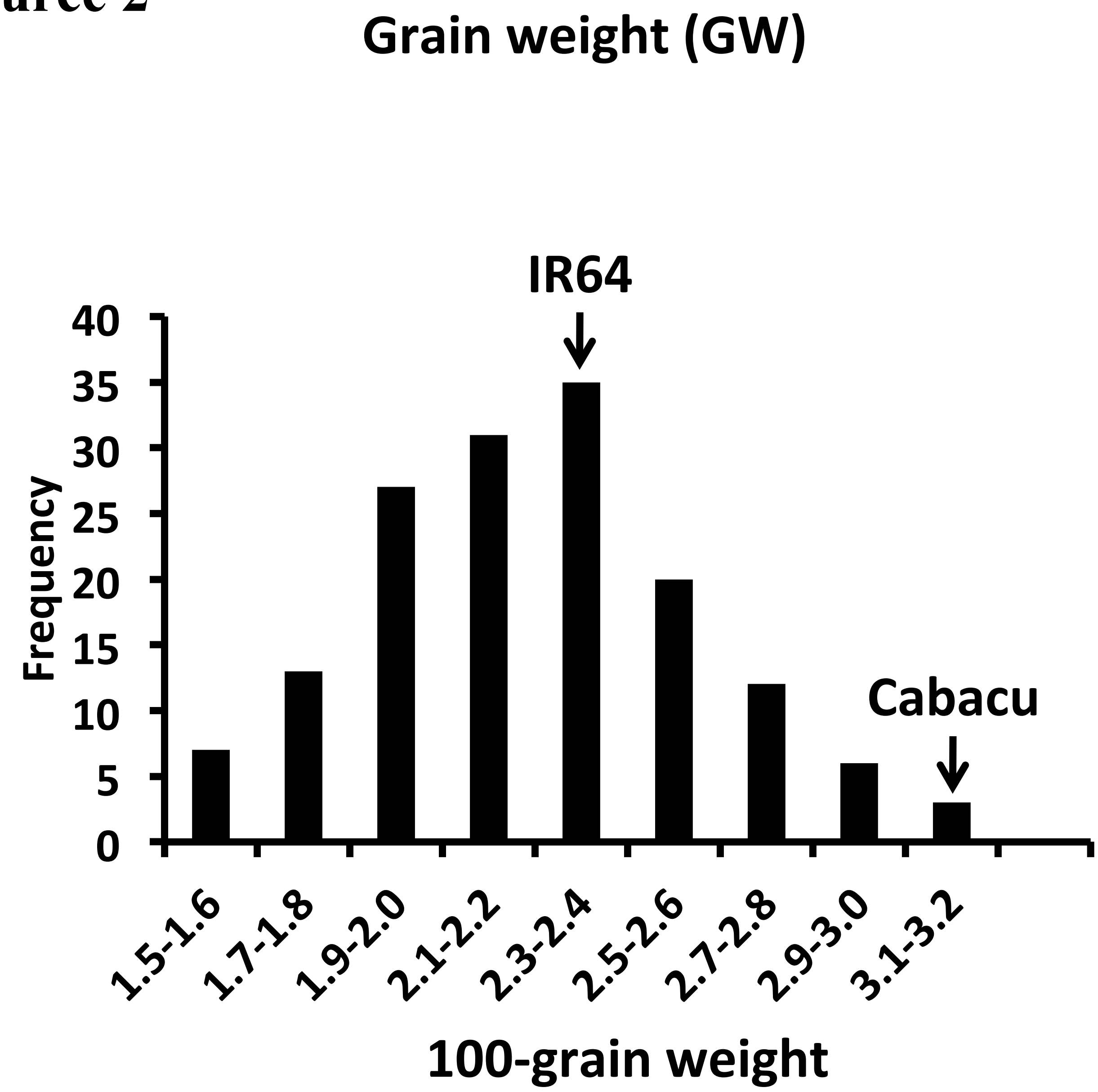

Online Resource 2

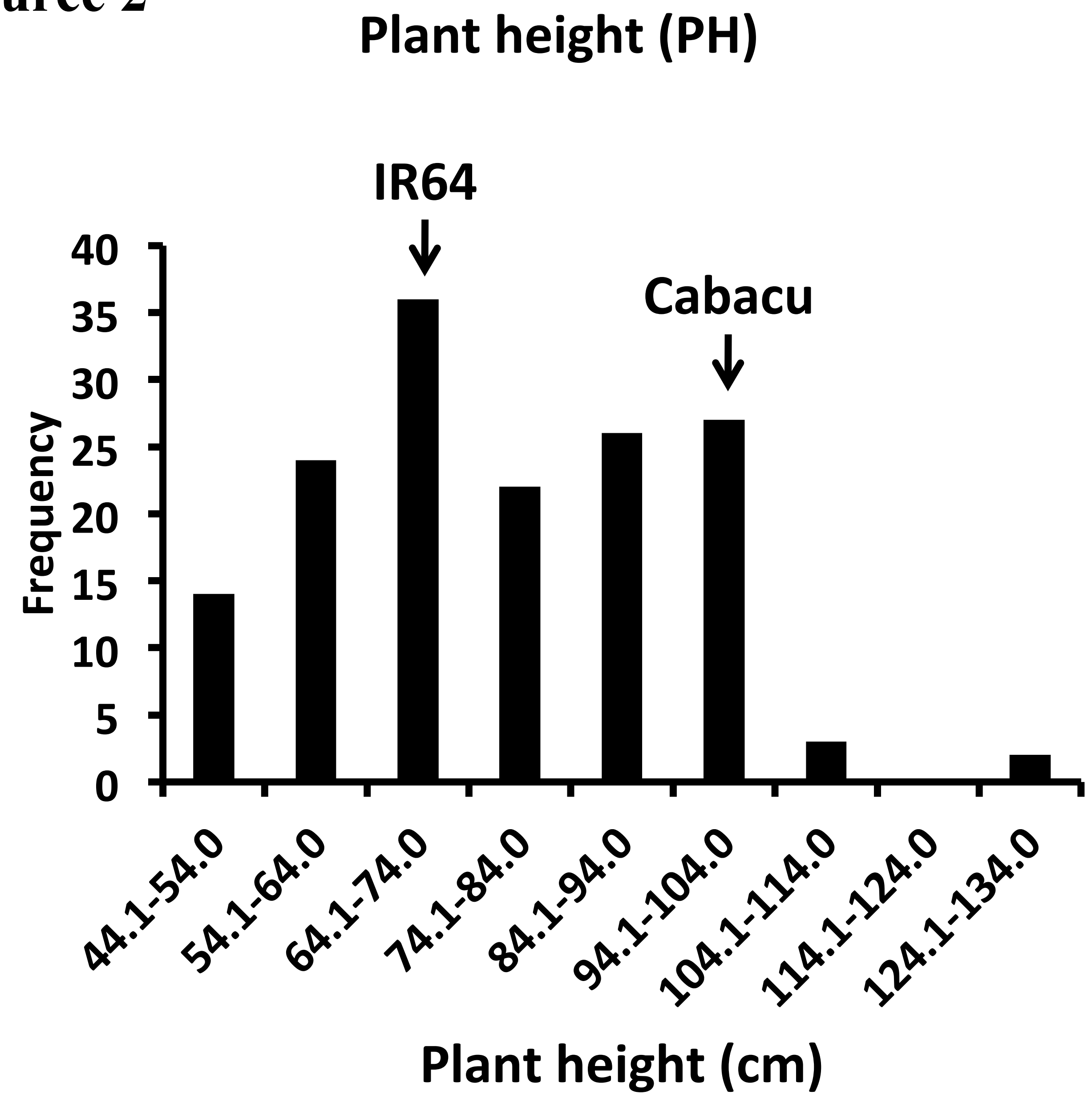

Online Resource 2

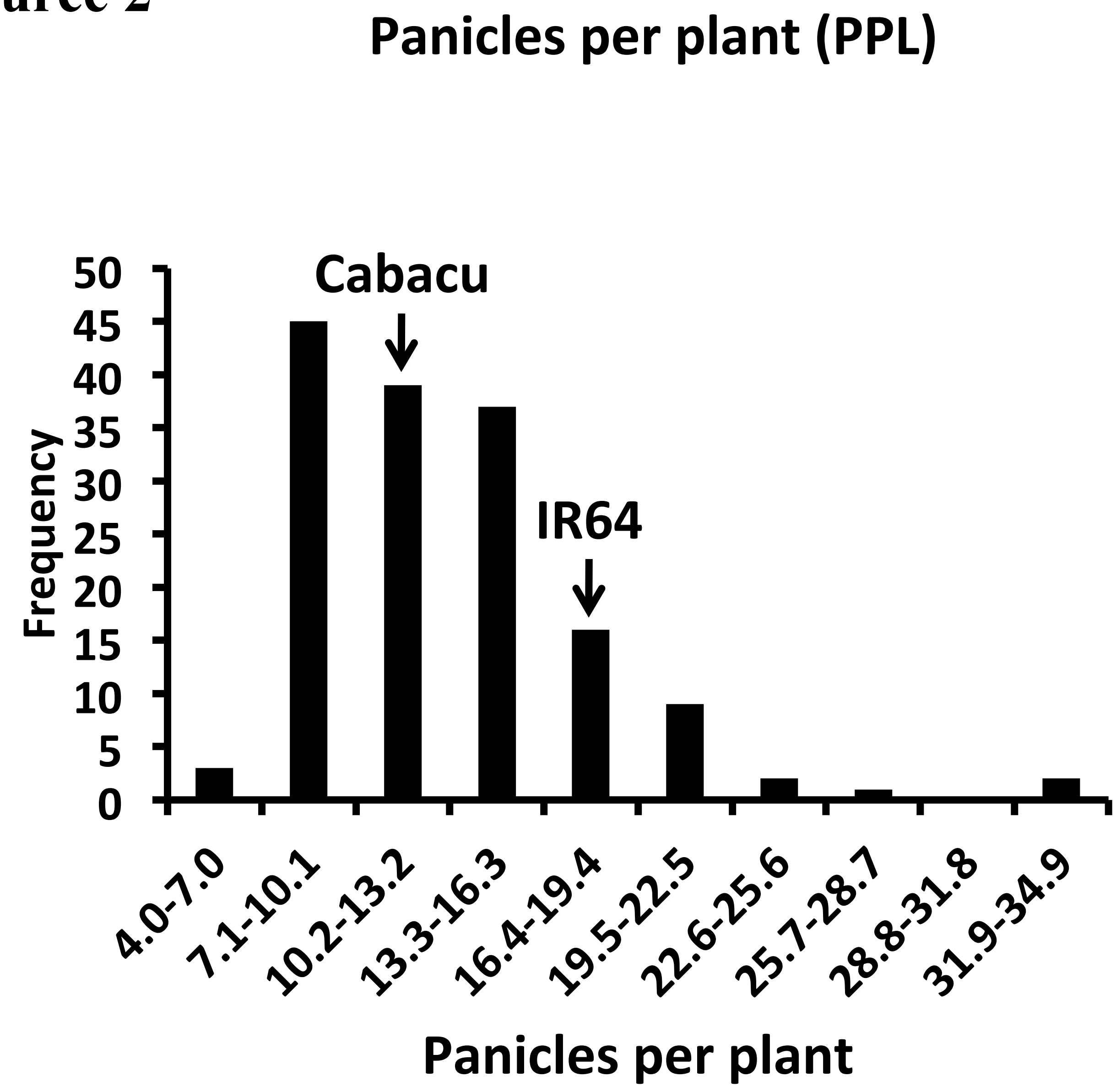

Online Resource 2

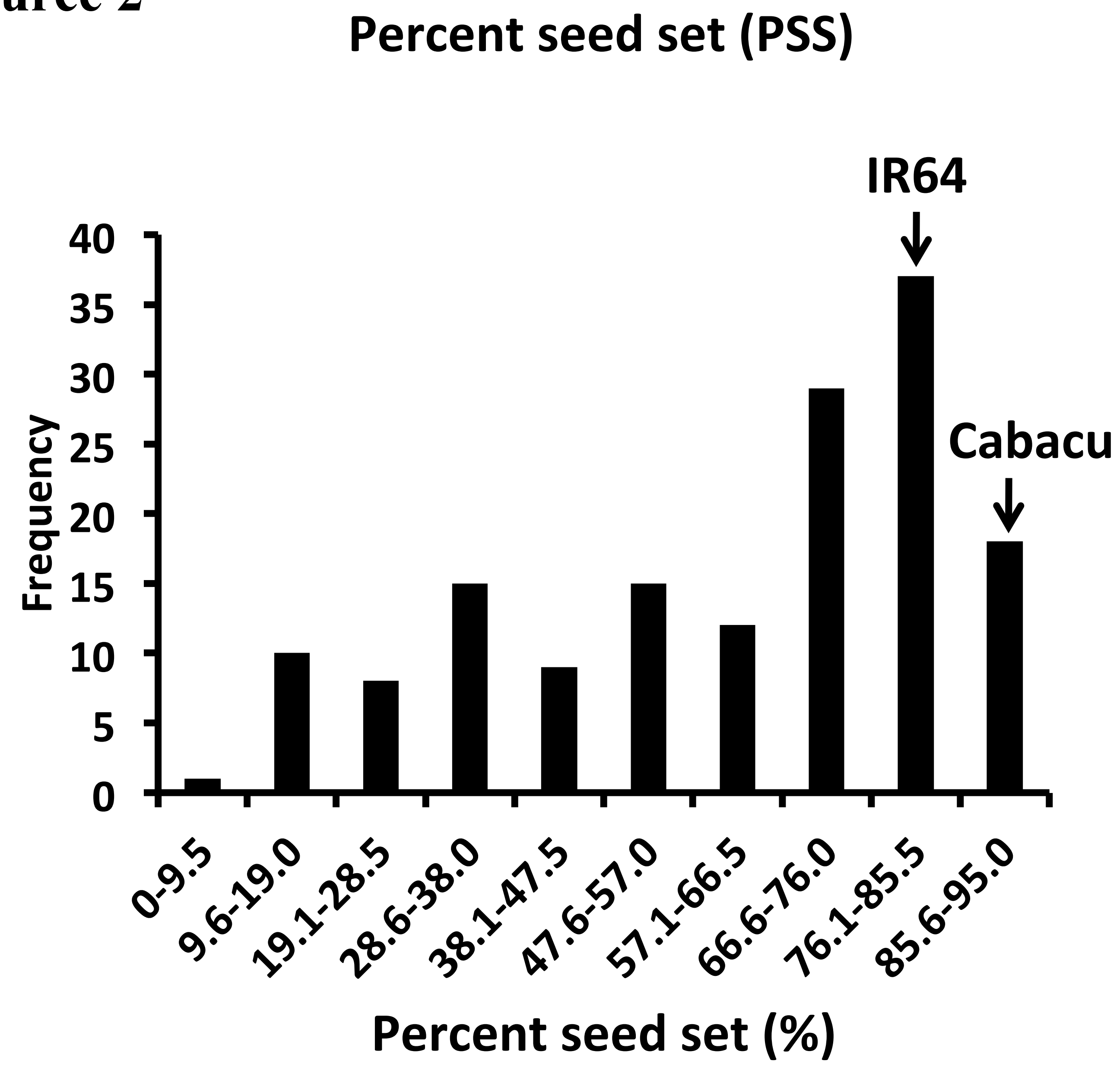

Online Resource 2

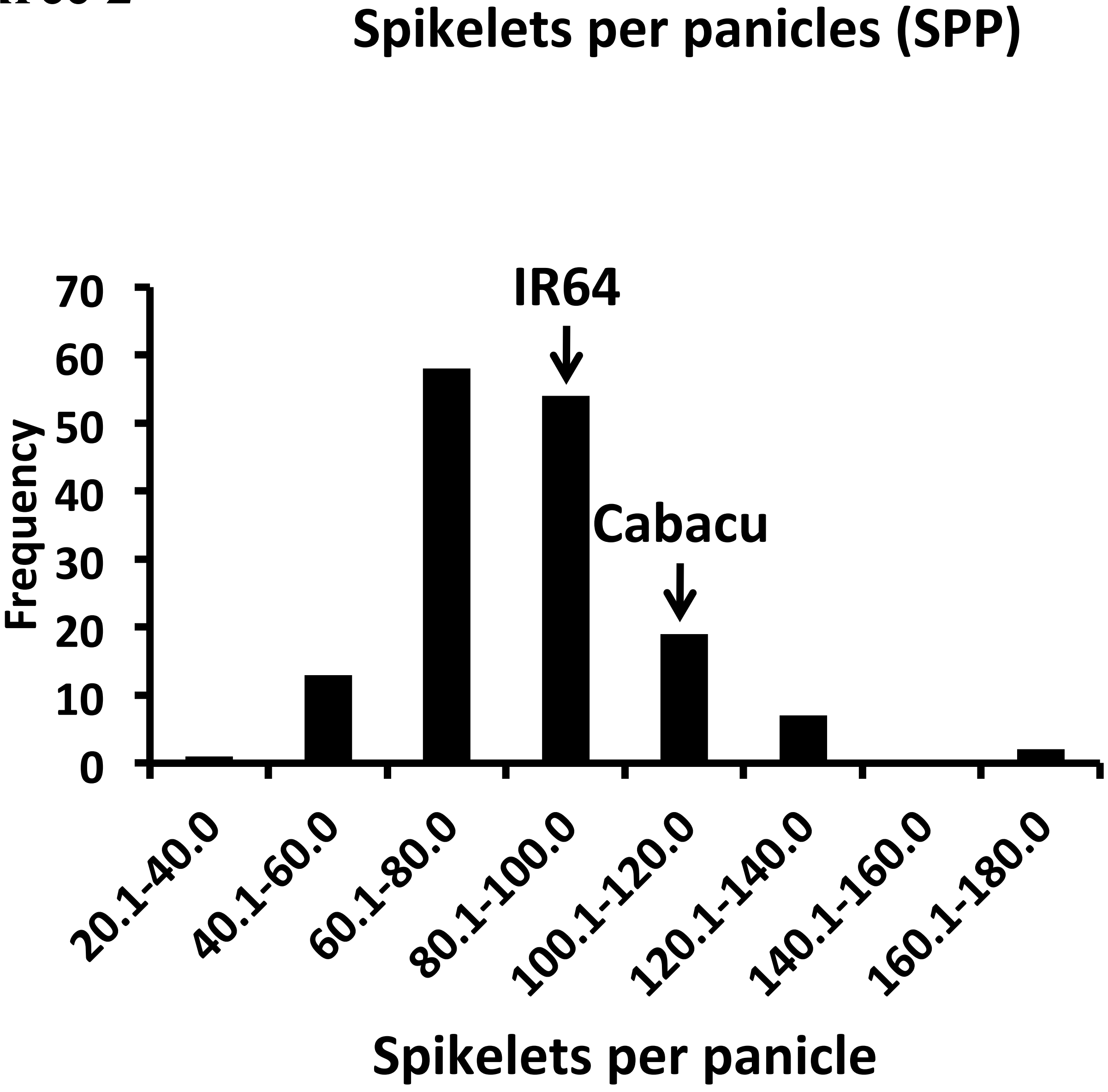

Supplement: Supplementary file 2 — Phenotypic distribution of drought stress responses under pre-flowering drought stress in a RIL population derived from a cross between IR64 and Cabacu. (PDF 664 kb) [file 11032_2013_12_MOESM2_ESM.pdf]
