# Supplementary material for: Meta-analysis of quantitative trait loci for grain yield and component traits under reproductive-stage drought stress in an upland rice population
Source: Mol Breed. 2014 Jun 29;34(2):283–95. doi: 10.1007/s11032-013-0012-0 (PMC4092238; doi:10.1007/s11032-013-0012-0)
Supplement: Supplementary file 4 — Table presents the distribution of polymorphic SNP markers on 12 rice chromosomes observed in this study. (PDF 69 kb) [file 11032_2013_12_MOESM4_ESM.pdf]

Online Resource 4 Molecular Breeding

Meta-analysis of QTLs for grain yield and component traits under reproductive-stage drought stress in an upland rice population.

Kurniawan R. Trijatmiko, Supriyanta, Joko Prasetiyono, Michael J. Thomson, Casiana M. Vera Cruz, Sugiono Moeljopawiro, Andy Pereira*.

*Crop, Soil & Environmental Sciences, University of Arkansas, Fayetteville, AR, USA; *apereira@uark.edu

Distribution of polymorphic SNP markers on 12 rice chromosomes observed in this study

Chr	∑ SNP marker	∑ polymorphic marker	Percentage of polymorphic marker (%)	Length of linkage map (cM)	Average of marker distance (cM)	Average of heterozygosity (%)
1	45	28	62.2	228.4	8.2	4.94
2	37	21	56.8	148.0	7.0	3.93
3	44	19	43.2	183.1	9.6	4.75
4	31	15	48.4	166.5	11.1	5.24
5	33	12	36.4	95.3	7.9	4.71
6	38	16	42.1	128.6	8.0	4.18
7	29	18	62.1	104.2	5.8	5.19
8	27	15	55.6	107.3	7.2	5.24
9	24	13	54.2	105.4	8.1	5.19
10	19	12	63.2	87.9	7.3	4.82
11	31	21	67.7	130.1	6.2	3.68
12	26	11	42.3	87.0	7.9	3.84
Total	384	201	Mean=52.3	Total=1571.8	Mean=7.9	Mean=4.63
