# Supplementary material for: Meta-analysis of quantitative trait loci for grain yield and component traits under reproductive-stage drought stress in an upland rice population
Source: Mol Breed. 2014 Jun 29;34(2):283–95. doi: 10.1007/s11032-013-0012-0 (PMC4092238; doi:10.1007/s11032-013-0012-0)

Online Resource 5 Molecular Breeding

Meta-analysis of QTLs for grain yield and component traits under reproductive-stage drought stress in an upland rice population.

Kurniawan R. Trijatmiko, Supriyanta, Joko Prasetiyono, Michael J. Thomson, Casiana M. Vera Cruz, Sugiono Moeljopawiro, Andy Pereira*.

*Crop, Soil & Environmental Sciences, University of Arkansas, Fayetteville, AR, USA; *apereira@uark.edu

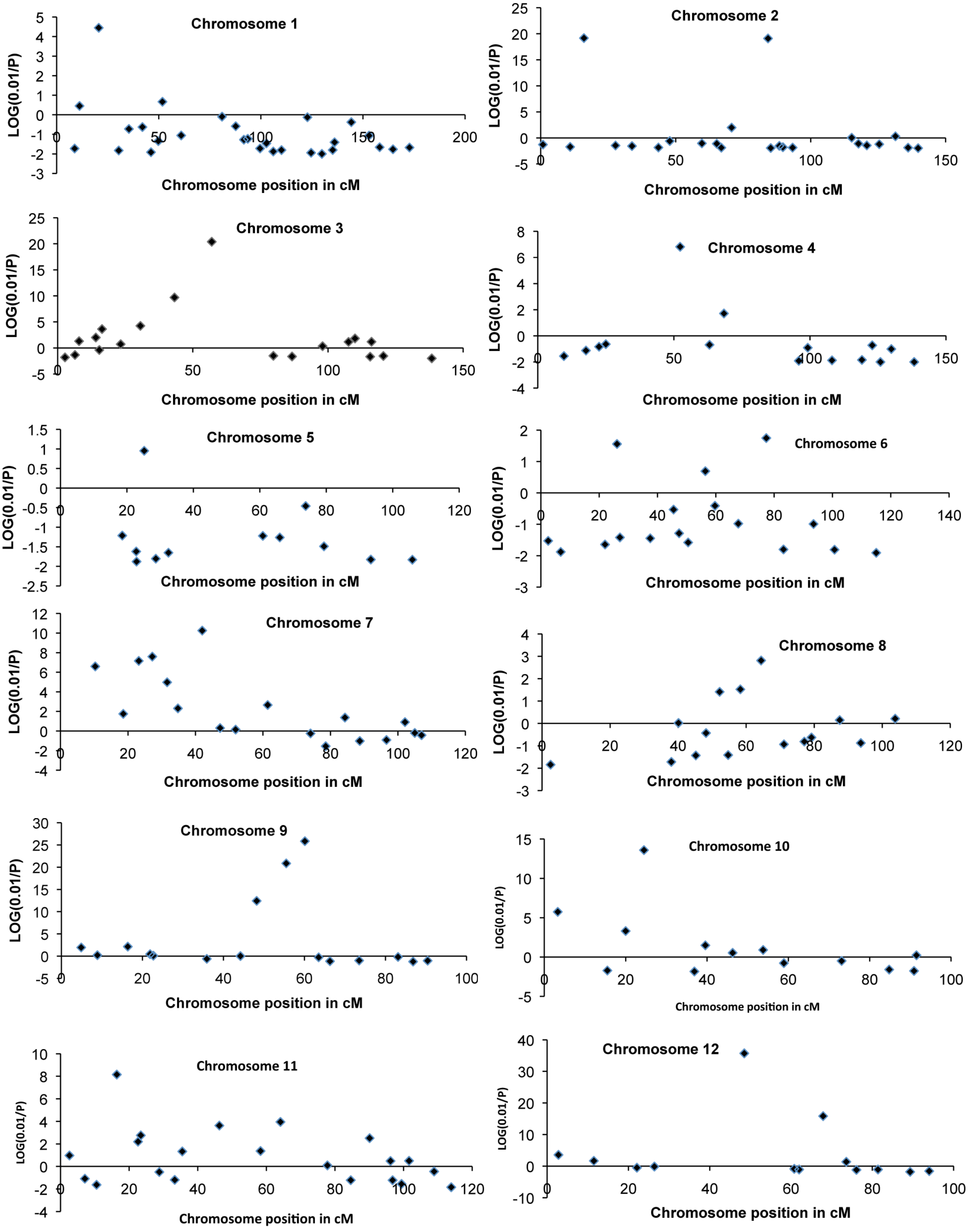

Supplement: Supplementary file 5 — Segregation distortion detected along the 12 rice chromosomes. The chromosomal position in centimorgans is on the x-axis, and LOG(0.01/P) values are on the y-axis. A value of LOG(0.01/P)>0 corresponds to P< 0.01. (PDF 315 kb) [file 11032_2013_12_MOESM5_ESM.pdf]
