# Supplementary material for: Meta-analysis of quantitative trait loci for grain yield and component traits under reproductive-stage drought stress in an upland rice population
Source: Mol Breed. 2014 Jun 29;34(2):283–95. doi: 10.1007/s11032-013-0012-0 (PMC4092238; doi:10.1007/s11032-013-0012-0)
Supplement: Supplementary file 6 — Table presents the comparative study of segregation distortion. (PDF 77 kb) [file 11032_2013_12_MOESM6_ESM.pdf]

Online Resource 6 Molecular Breeding
Meta-analysis of QTLs for grain yield and component traits under reproductive-stage drought stress in an upland rice population.

Kurniawan R. Trijatmiko, Supriyanta, Joko Prasetiyono, Michael J. Thomson, Casiana M. Vera Cruz, Sugiono Moeljopawiro, Andy Pereira*.
*Crop, Soil & Environmental Sciences, University of Arkansas, Fayetteville, AR, USA; *apereira@uark.edu

Comparative study of segregation distortion

Chr	Position (cM)	P-value	Gametophyte or sterility genes	Previous studies with common distorted regions	Population	Direction of skewness	
						Current study	Previous study
1	27.8	< 0.001	*ga-9*	Xu et al. 1997	BC	IR64	*indica*
3	7.6	< 0.01	*ga-2*, *ga-3*	Xu et al. 1997	BC	IR64	*indica*
3	16.8-86	< 0.001	*ga-2*, *ga-3*	Xu et al. 1997	BC, F_2	IR64	*indica*
3	135.1	< 0.01	*S-9*	Xu et al. 1997	RIL	IR64	*indica*
4	23.3	< 0.001	*s-e-2*, *ga-6*, *ga-10*	Lin et al. 1998	BC_1F_5	Cabacu	*japonica*
7	11.0-71.6	< 0.001	*ga-11*, *S-7*	Xu et al. 1997	F_2, DH	IR64	*indica*
7	104.8	< 0.001		Xu et al. 1997	RIL	Cabacu	*japonica*
8	55.4-64.4	< 0.001		Xu et al. 1997	DH	IR64	*indica*
9	0.8	< 0.01		Xu et al. 1997	DH	IR64	*indica*
9	3.2	< 0.001		Xu et al. 1997	DH, RIL	IR64	*indica*
9	63.0	< 0.001		Xu et al. 1997	RIL	IR64	*indica*
10	9.5-13.3	< 0.01		-	-	IR64	-
10	15.2	< 0.001		Harushima et al. 1996	F_2	Cabacu	*indica*
10	35.9	< 0.01		Harushima et al. 1996	F_2	IR64	*indica*
11	21.3-37.3	< 0.001		Xu et al. 1997	DH	IR64	*indica*
11	50.6-55.9	< 0.001	*s-3*	Xu et al. 1997	DH	IR64	*indica*
11	64.2	< 0.001		Xu et al. 1997	DH	IR64	*indica*
11	96.4	< 0.01		Xu et al. 1997	DH	IR64	*indica*
11	103.9-106.8	< 0.001		Xu et al. 1997	DH	IR64	*indica*
12	9.5	< 0.001		Xu et al. 1997	RIL	IR64	*indica*
