# Supplementary material for: Meta-analysis of quantitative trait loci for grain yield and component traits under reproductive-stage drought stress in an upland rice population
Source: Mol Breed. 2014 Jun 29;34(2):283–95. doi: 10.1007/s11032-013-0012-0 (PMC4092238; doi:10.1007/s11032-013-0012-0)
Supplement: Supplementary file 8 — Table presents QTLs for percent seed set, grains per panicle and plant height under drought condition identified in the IR64/Cabacu RIL population and subpopulations with fixed alleles at the sd1 locus (PDF 80 kb) [file 11032_2013_12_MOESM8_ESM.pdf]

Online resource 8 Molecular Breeding

Meta-analysis of QTLs for grain yield and component traits under reproductive-stage drought stress in an upland rice population.

Kurniawan R. Trijatmiko, Supriyanta, Joko Prasetiyono, Michael J. Thomson, Casiana M. Vera Cruz, Sugiono Moeljopawiro, Andy Pereira*.

*Crop, Soil & Environmental Sciences, University of Arkansas, Fayetteville, AR, USA; *apereira@uark.edu

QTLs for percent seed set, grains per panicle and plant height under drought condition identified in the IR64/Cabacu RIL population and subpopulations with fixed alleles at the *sd1* locus

Traits[a]	Subpopulation	QTLs	Chr	Peak marker	Increased effect	LOD	R^2 (%)[b]	A[c]
PSS	Whole	*qPSS8.1*	8	id8003838	IR64	**5.3**[d]	14.7	9.2
	sd1	-[f]						
	SD1	*qPSS8.1*	8	id8003838	IR64	**4.1**	24.5	13.0
	Whole	*qPSS8.2*	8	id8005359	IR64	**7.7**	20.5	11.3
	sd1	-						
	SD1	*qPSS8.2*	8	id8005359	IR64	**4.7**	27.8	14.5
GPP	Whole	*qGPP8.2*	8	id8005359	IR64	**6.0**	16.4	10.8
	sd1	-						
	SD1	*qGPP8.2*	8	id8005359	IR64	3.7[e]	22.4	12.2
PH	Whole	-						
	sd1	-						
	SD1	*qPH5.1*	5	id5007714	IR64	**3.9**	23.4	8.0
	Whole	-						
	sd1	*qPH11.1*	11	id11000858	Cabacu	**3.9**	24.0	-6.4
	SD1	-						

[a]Abbreviations: PSS percent seed set, GPP grains per panicle, PH plant height

[b]Relative contributions of the putative QTLs to the phenotypic variation

[c]Additive effect. The positive or negative value indicates that allele from IR64 or Cabacu increases the trait value, respectively

[d]QTLs in bold face were identified at $P < 0.01$ using permutation analysis

[e]QTLs in regular type were identified at $P < 0.05$ using permutation analysis

[f]No QTL was identified at $P < 0.05$ using permutation analysis
